# Contribution of microglia and astrocytes to the central sensitization, inflammatory and neuropathic pain in the juvenile rat

**DOI:** 10.1186/1744-8069-8-43

**Published:** 2012-06-15

**Authors:** Hiroshi Ikeda, Takaki Kiritoshi, Kazuyuki Murase

**Affiliations:** 1 Department of Human and Artificial Intelligence Systems, Graduate School of Engineering; Research and Education Program for Life Science, University of Fukui, 3-9-1 Bunkyo, Fukui, 910-8507, Japan

**Keywords:** Plasticity, Hyperalgesia, Optical imaging, Calcium imaging

## Abstract

**Background:**

The development of pain after peripheral nerve and tissue injury involves not only neuronal pathways but also immune cells and glia. Central sensitization is thought to be a mechanism for such persistent pain, and ATP involves in the process. We examined the contribution of glia to neuronal excitation in the juvenile rat spinal dorsal horn which is subjected to neuropathic and inflammatory pain.

**Results:**

In rats subjected to neuropathic pain, immunoreactivity for the microglial marker OX42 was markedly increased. In contrast, in rats subjected to inflammatory pain, immunoreactivity for the astrocyte marker glial fibrillary acidic protein was increased slightly. Optically-recorded neuronal excitation induced by single-pulse stimulation to the dorsal root was augmented in rats subjected to neuropathic and inflammatory pain compared to control rats. The bath application of a glial inhibitor minocycline and a p38 mitogen-activated protein kinase inhibitor SB203580 inhibited the neuronal excitation in rats subjected to neuropathic pain. A specific P2X_1,2,3,4_ antagonist TNP-ATP largely inhibited the neuronal excitation only in rats subjected to neuropathic pain rats. In contrast, an astroglial toxin L-alpha-aminoadipate, a gap junction blocker carbenoxolone and c-Jun N-terminal kinase inhibitor SP600125 inhibited the neuronal excitation only in rats subjected to inflammatory pain. A greater number of cells in spinal cord slices from rats subjected to neuropathic pain showed Ca^2+^ signaling in response to puff application of ATP. This Ca^2+^ signaling was inhibited by minocycline and TNP-ATP.

**Conclusions:**

These results directly support the notion that microglia is more involved in neuropathic pain and astrocyte in inflammatory pain.

## Background

Persistent pain caused by peripheral nerve injury (neuropathic pain) and peripheral tissue injury (inflammation) increases the sensitivity to noxious stimuli (hyperalgesia) and/or induces a pain sensation in response to light-touch (allodynia). Central sensitization, which is an enhanced responsiveness of nociceptive neurons in the central nervous system to their normal afferent input, is thought to be a mechanism for hyperalgesia and allodynia [1,2].

In recent years, it is increasingly recognized that glial cells in the spinal dorsal horn, such as microglia and astrocytes, are activated in response to peripheral nerve injury and peripheral tissue injury and are involved in spinal nociceptive transmission and central sensitization [3-5]. Activation of glial cells has been shown to be directly involved in neuropathic and inflammatory pain, since blocking the activation of spinal cord microglia with minocycline [6-8] and astrocytes with fluorocitrate and L-alpha-aminoadipate [9,10] prevents or delays the development of allodynia and hyperalgesia.

A number of mechanisms for the induction and maintenance of glia-related persistent pain have been suggested. These include activation of mitogen-activated protein kinases (MAPKs), such as the extracellular signal-regulated kinases (ERKs) [11,12], p38 MAPK [13,14] and c-Jun N-terminal kinases (JNK) [9,11,15], upregulated expression of P2 purinoceptors [16], and increased proinflammatory cytokines such as interleukin (IL)-1β, IL-6, tumor necrosis factor (TNF)-α and IL-18 [13,17], and of chemokines such as CCL2 [18]. How these kinases and purinergic receptors affect the electrophysiological activities in the spinal cords has also been reported [18,19]. The responsiveness of glial cells to ATP in neuropathic pain condition is also revealed [14,19,20].

However, there is no direct evidence which compare the amplitude of neuronal excitation and the effect of glia-related agents on the excitation under persistent pain induced by peripheral nerve and tissue injury with control, untreated condition. The comparison of Ca^2+^ signal to ATP stimulation in the spinal slices under the condition of inflammatory or neuropathic pain with control condition is also not reported. The aim of this study is to reveal whether or not the glia-related agents, which have been used in various studies, are indeed affecting the neuronal excitation recorded by optical method. More specifically, we focus on whether microglia and astrocyte have differential roles in neuropathic pain and inflammatory pain. We also like to study how ATP induces Ca^2+^ signals in the superficial dorsal horn in slices obtained under neuropathic and inflammatory conditions.

## Results

### Morphological changes of glial cells in the spinal dorsal horn in response to inflammatory and neuropathic pain

**Figure 1:**
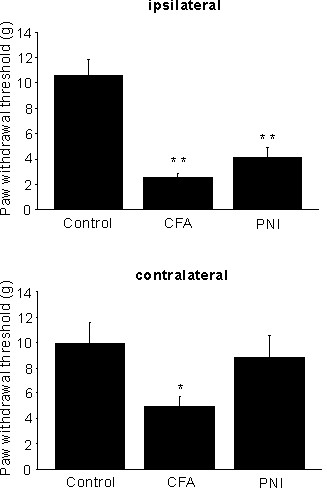
**Mechanical allodynia produced by unilateral injection of CFA or by PNI in rats.** Mechanical allodynia produced by unilateral injection of complete Freund adjuvant (CFA) or by peripheral nerve injury (PNI) in rats. Rats were tested for mechanical sensitivity of ipsilateral (ipsi) and contralateral (contra) hind paws 1 week after treatment. Data are expressed as mean withdrawal threshold (g). *P < 0.05, **P < 0.01.

**Figure 2:**
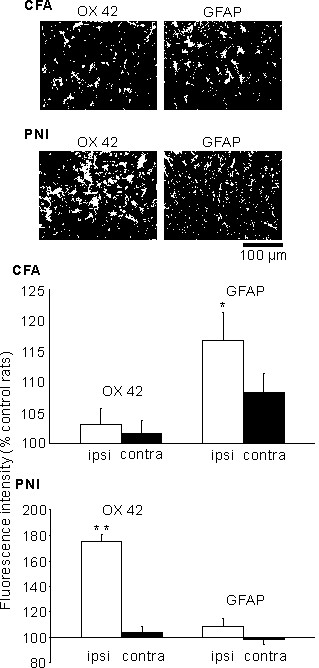
**Quantification of OX 42 and glial fibrillary acidic protein (GFAP) immunofluorescence intensity.** Quantification of OX 42 and glial fibrillary acidic protein (GFAP) immunofluorescence intensity in the ipsilateral (ipsi; white bars) and contralateral (contra; black bars) spinal dorsal horn of rats injected with complete Freund adjuvant (CFA) or rats subjected to peripheral nerve injury (PNI). Data are expressed as % of mean intensity of OX 42 and GFAP immunofluorescence in control, untreated rats. *P < 0.05, **P < 0.01.

To examine morphologic changes of glial cells during pain hypersensitivity under condition of inflammatory pain and neuropathic pain, we performed the immunohistochemical staining of glial cells. One week after unilateral injection of CFA into the hind paw (used as a model of inflammatory pain), the paw-withdrawal threshold to mechanical stimulation was significantly decreased in the ipsilateral and contralateral paw in comparison to control, untreated rats (CFA: n = 16, control: n = 8, ipsilateral: P < 0.01, contralateral: P < 0.05) (Figure 1). Quantification of immunoreactivity by measuring the intensity of the staining revealed a significant increase in GFAP immunoreactivity (astrocyte marker), but not in OX 42 immunoreactivity (microglia marker), in ipsilateral and contralateral superficial dorsal horn (n = 16, GFAP: ipsilateral, 117 ± 5%, P < 0.05, contralateral, 108 ± 3%, P = 0.1, OX42: ipsilateral, 103 ± 3%, P = 0.9, contralateral, 102 ± 3%, P = 0.9) (Figure 2). The immunoreactivity of GFAP and OX42 was not increased in saline-injected rats (n = 3, GFAP: ipsilateral, 104 ± 5%, P = 0.8, contralateral, 99 ± 1%, P = 0.9, OX42: ipsilateral, 97 ± 1%, P = 0.8, contralateral, 99 ± 2%, P = 0.9).

One week after unilateral PNI of the fifth lumber spinal nerves (used as a model of neuropathic pain), the paw-withdrawal threshold to the mechanical stimulation was significantly decreased in the ipsilateral but not contralateral paw (n = 6, ipsilateral: P < 0.01, contralateral: P = 0.6) (Figure 1). Quantification of immunoreactivity revealed a significant increase in OX 42 immunoreactivity, but not in GFAP immunoreactivity, on the ipsilateral superficial dorsal horn, and there was no significant change in OX 42 or GFAP immunoreactivity on the contralateral side (n = 14, GFAP: ipsilateral, 109 ± 6%, P = 0.8, contralateral, 98 ± 4%, P = 0.9, OX42: ipsilateral, 175 ± 5%, P < 0.01, contralateral, 104 ± 5%, P = 0.9) (Figure 2). The immunoreactivity of GFAP and OX42 was not increased in sham-operated rats (n = 4, GFAP: ipsilateral, 102 ± 4%, P = 0.9, contralateral, 101 ± 9%, P = 0.9, OX42: ipsilateral, 122 ± 10%, P = 0.2, contralateral, 98 ± 4 %, P = 0.9).

### Difference in optically-recorded neuronal excitation evoked by dorsal root stimulation

**Figure 3:**
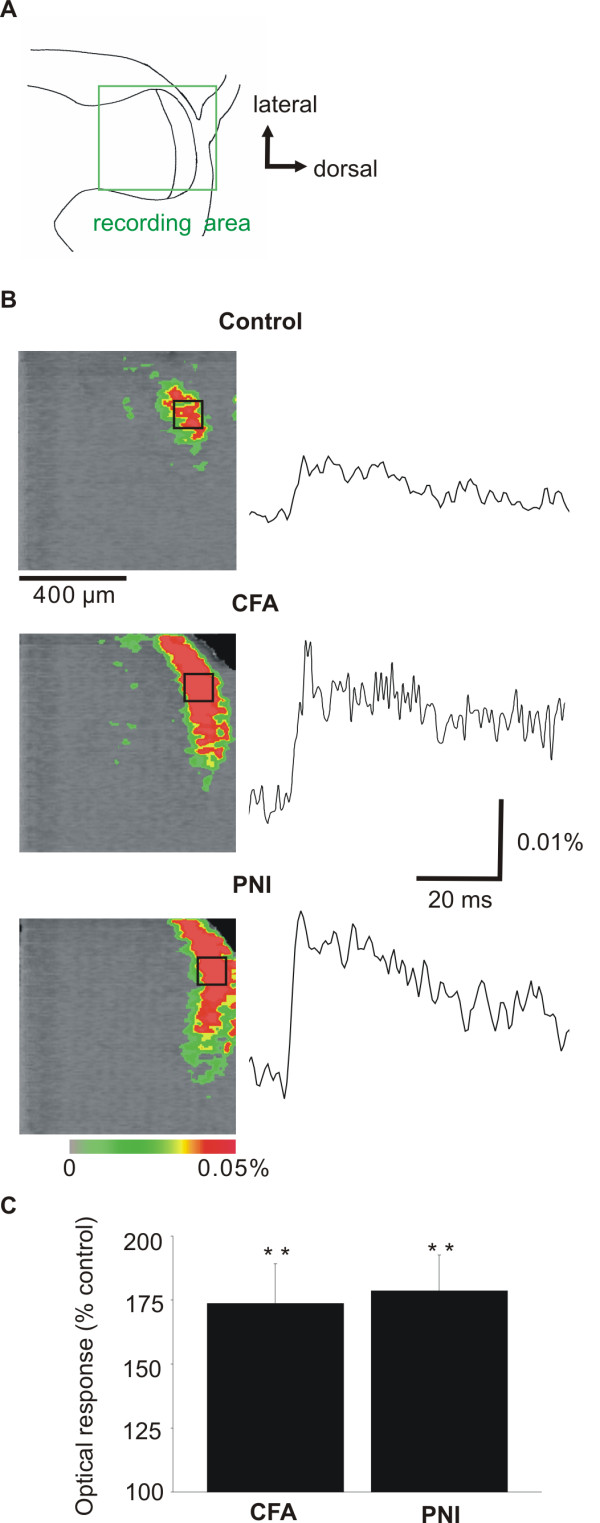
**Neuronal excitation in the spinal cord slices.** Neuronal excitation in the spinal cord slices in control rats, rats injected with complete Freund adjuvant (CFA), and rats subjected to peripheral nerve injury (PNI). **A**, Schematic of a transverse spinal cord slice. Images in **B** were obtained from the area indicated by the green rectangle in **A**. **B,** Images in the left column are representative examples of optical responses elicited by a single-pulse stimulation to the dorsal root at the peak time point. The percent change in light absorption is depicted by simulated color, as indicated by the color bar. Graphs in the right column are representative examples of time courses of optical responses. Each time course was indicate examples of time courses of optical responses. Each time course was an average of 128 optical responses recorded by 16 × 16 pixels on the image sensor in the dorsal horn indicated by the black rectangle in each image. Stimulus pulses were administered at the time indicated by the filled triangle. **C,** Mean intensity of optical response. The intensity of each response was obtained by temporal integration of optical response magnitude during the 18-ms period after the onset of response. Data are expressed as % of mean intensity of optical response in control, untreated rats. *P < 0.05, **P < 0.01.

We visualized gross neuronal excitation in the superficial dorsal horn of transverse spinal cord slices stained with the voltage-sensitive dye, RH-482 [21-23]. A single-pulse stimulation of C fiber-activating strength to the dorsal root evoked an optical response in spinal laminae I-III (Figure 3B), representing excitation of neurons throughout the thickness of the slice. Neuronal excitation in the ipsilateral spinal dorsal horn evoked by such stimulus was augmented in CFA injected rats (174 ± 16%, n = 20, P < 0.01) and in rats subjected to PNI (178 ± 14 %, n = 10, P < 0.01) in comparison to control, untreated rats (Figure 3B, C). There were no significant difference in optically recorded neuronal excitation in slices obtained from CFA injected rats and rats subjected to PNI (P = 0.9). These augmentations of neuronal excitation in CFA injected rats and rats subjected to PNI were observed not only in L5 injured segment of spinal cord, but also in neighboring uninjured segments (L4, L6). The magnitude of A-fiber response induced by low-intensity stimulation was usually 0.01% or less of the background light intensity, close to the noise level of the system [21]. Therefore, we did not examine optical responses induced by low-intensity stimulation.

### Contribution of microglia, purinoceptors and p38 MAPK to hyperexcitability of the spinal dorsal horn by neuropathic pain

It has been reported that the expression of the P2X_4_ receptor subtype of ionotropic purinoceptor and activation of p38 MAPK increase in microglia, but not neurons or astrocytes, in the ipsilateral spinal dorsal horn after PNI, and that this increase parallels the increase in pain hypersensitivity [12,14,17]. We therefore examined the contributions of microglia, the P2X_4_ receptor, and p38 MAPK to the augmentation of neuronal excitation in the superficial spinal dorsal horn of rats injected with CFA and rats subjected to PNI.

**Figure 4:**
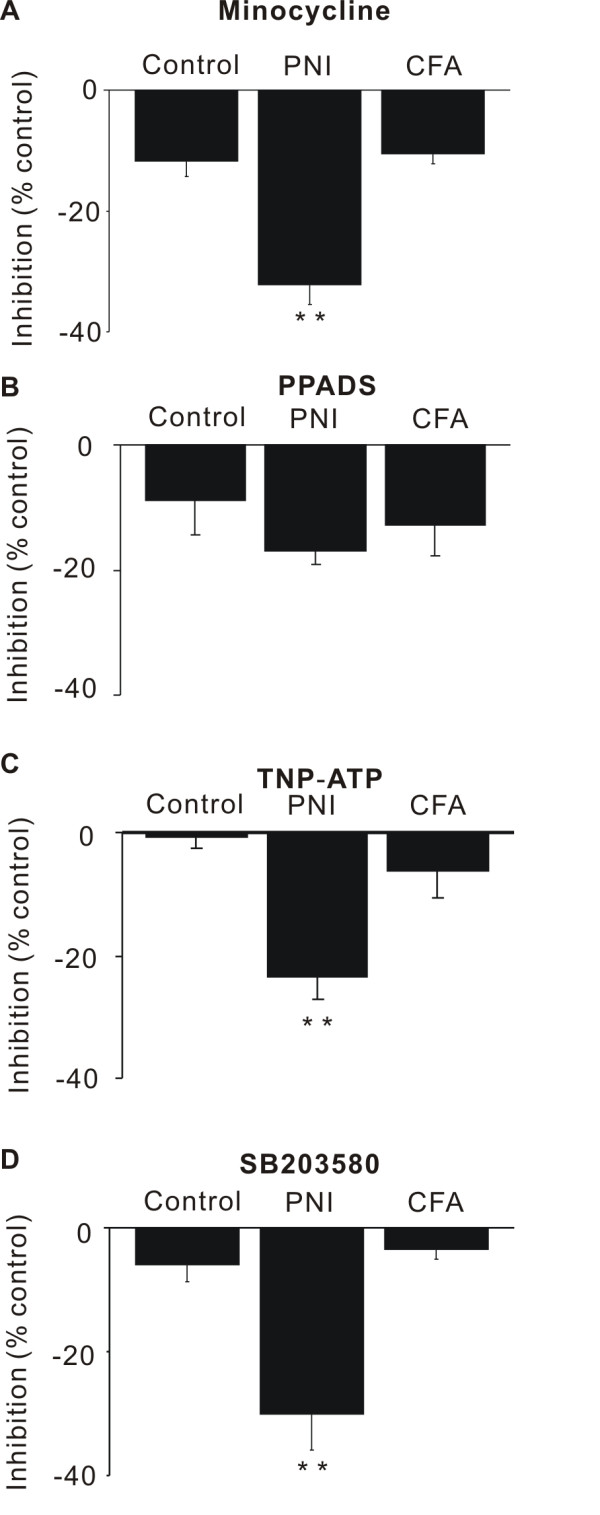
**Effect of microglial inhibitor, P2X receptor antagonists and p38 MAPK inhibitor on neuronal excitation.** Effect of the microglial inhibitor minocycline (20 μM) (**A**), the specific P2X_1,2,3,5_ antagonist pyridoxal-phosphate-6-azophenyl-2',4'-disulfonate (PPADS, 20 μM) (**B**), the specific P2X_1,2,3,4_ antagonist 2',3'-O-(2,4,6-trinitrophenyl) adenosine 5'-triphosphate (TNP-ATP, 1 μM) (**C**) and the p38 MAPK inhibitor SB203580 (1 μM) (**D**) on neuronal excitation in control rats, rats injected with complete Freund adjuvant (CFA), and rats subjected to peripheral nerve injury (PNI). Data are expressed as % inhibition of the mean intensity of the optical response during the preincubation period. *P < 0.05, **P < 0.01.

Neuronal excitation in rats subjected to PNI was significantly inhibited by the microglial inhibitor minocycline (20 μM, 30 min) in comparison to control, untreated rats (-32 ± 3%, *n* = 7, P < 0.01), whereas that in rats injected with CFA was unaffected (-11 ± 2%, n = 6, P = 0.7) (Figure 4A). Neuronal excitation in both group of rats was not significantly inhibited by the specific P2X_1,2,3,5_ antagonist pyridoxal-phosphate-6-azophenyl-2',4'-disulfonate (PPADS) (20 μM, 30min) in comparison to control rats (PNI: -17 ± 2%, n = 5, P = 0.1: CFA: -13 ± 5 %, n = 6, P = 0.5) (Figure 4B). However, neuronal excitation in rats subjected to PNI was significantly inhibited by a mixed solution of PPADS and the specific P2X_1,2,3,4_ antagonist 2',3'-O-(2,4,6-trinitrophenyl) adenosine 5'-triphosphate (TNP-ATP) (1 μM, 30 min) after incubation with PPADS alone (PNI: -23 ± 4%, *n* = 5, P < 0.01, CFA: -6 ± 4%, n = 6, P = 0.3) (Figure 4C). Neuronal excitation in rats subjected to PNI was also inhibited by p38 MAPK inhibitor SB203580 (1 μM, 30 min) (PNI: -30 ± 3%, n = 5, P < 0.01, CFA: -4 ± 6%, *n* = 3, P = 0.7) (Figure 4D). These results indicate that hyperexcitability in rats subjected to PNI are contributed by microglia and P2X_4_ and p38 MAPK.

### Contribution of JNK and gap junction in astrocyte to hyperexcitability in the spinal dorsal horn by inflammatory insult, but by nerve injury

**Figure 5:**
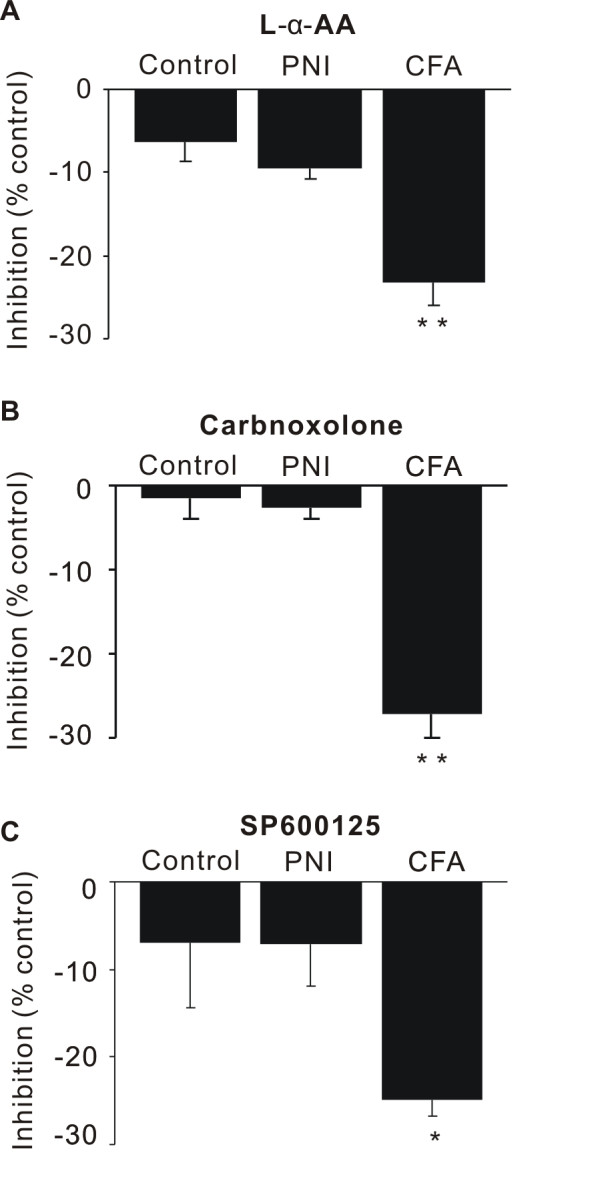
**Effect of astrocyte toxin, gap junction blocker and JNK inhibitor on neuronal excitation.** Effect of the astroglial toxin L-alpha-aminoadipate (L-α-AA, 1 mM) (**A**), a gap junction blocker carbenoxolone (100 μM) (**B**) and the JNK inhibitor SP600125 (10 μM) (**C**) on neuronal excitation in control rats, rats injected with complete Freund adjuvant (CFA), and rats subjected to peripheral nerve injury (PNI). Data are expressed as % inhibition of the mean intensity of optical response during the preincubation period. *P < 0.05, **P < 0.01.

Astrocytes in the spinal dorsal horn also play an important role in pain hypersensitivity. It has been reported that activation of astrocyte, JNK and gap junctions contributes to pain hypersensitivity [9,11,15,24]. We therefore examined the contribution of astrocytes, namely whether or not JNK and gap junction contribute to the augmentation of neuronal excitation in the superficial spinal dorsal horn of rats injected with CFA and rats subjected to PNI. Neuronal excitation in rats injected with CFA, but not in rats subjected to PNI, was significantly inhibited by the astroglial toxin L-alpha-aminoadipate (L-α-AA, 1 mM, 20 min) (PNI: -10 ± 1%, n = 5, P = 0.3, CFA: -25 ± 2 %, n = 4, P < 0.01), a gap junction blocker carbenoxolone (100 μM, 20 min, PNI: -10 ± 1%, *n* = 5, P = 0.9, CFA: -27 ± 5%, n = 4, P < 0.01) and the JNK inhibitor SP600125 (10 μM, 30 min, PNI: -3 ± 2%, n = 5, P = 1, CFA: -23 ± 3%, *n* = 10, P < 0.05) in comparison to control rats (Figure 5).

### Augmentation of ATP-induced Ca^2+^ signaling via P2X_4_ receptors in the spinal dorsal horn by nerve injury

Glial cells are not electrically activated and do not communicate directly via action potentials. However, they display a form of excitability that is manifested by an increase in intracellular Ca^2+^ concentration [25,26]. We therefore visualized increases in intracellular Ca^2+^ by Ca^2+^ imaging in the spinal cord slices.

**Figure 6:**
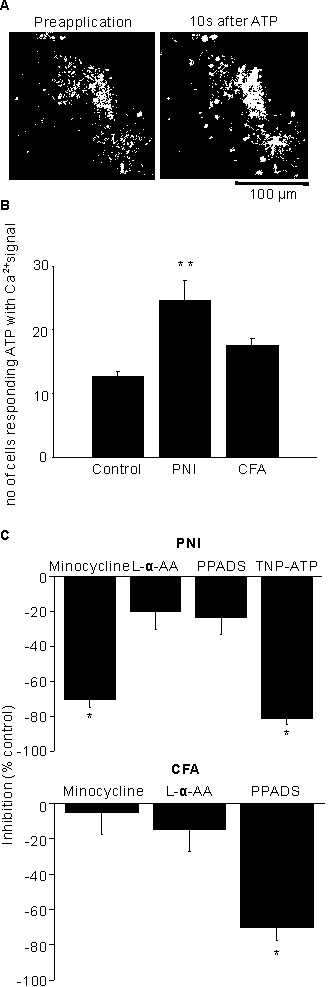
**Ca**^**2+**^**signals induced by puff-application of ATP.** Ca^2+^ signals induced by puff-application of ATP. **A**, Representative example of Ca^2+^ signals in the spinal dorsal horn of a rat subjected to peripheral nerve injury (PNI) and treated with puff-application of ATP. White dots in the left image indicate cells in the spinal dorsal horn loaded with Fluo-4/AM during preapplication of ATP. The right image shows the Ca^2+^ signal response to puff-application of ATP 10 s after application. **B**, Quantification of the number of cells showing a Ca^2+^ signal response to ATP in control rats, rats injected with complete Freund adjuvant (CFA), and rats subjected to PNI. *P < 0.05, **P < 0.01 compared to control rats. **C**, The upper graph indicates the effect of the microglial inhibitor minocycline (20 μM), the specific P2X_1,2,3,5_ antagonist pyridoxal-phosphate-6-azophenyl-2',4'-disulfonate (PPADS, 20 μM), the specific P2X_1,2,3,4_ antagonist 2',3'-O-(2,4,6-trinitrophenyl) adenosine 5'-triphosphate (TNP-ATP, 1 μM) on Ca^2+^ signals in rats subjected to PNI and responding to ATP. The lower graph indicates the effect of the microglial inhibitor minocycline (20 μM), the astroglial toxin L-alpha-aminoadipate (L-α-AA, 1 mM) and the specific P2X_1,2,3,5_ antagonist pyridoxal-phosphate-6-azophenyl-2',4'-disulfonate (PPADS, 20 μM) on Ca^2+^ signals in rats subjected to CFA and responding to ATP. Data are expressed as % of the decrease in the number of cells responding to drug application. *P < 0.05, **P < 0.01

A greater number of cells in the ipsilateral spinal dorsal horn responded to puff -application of ATP (100 μM) with increased Ca^2+^ signaling in rats subjected to PNI than in control rats (25 ± 3 cells vs. 13 ± 1 cells, respectively, n = 21, P < 0.01). This was not observed in rats injected with CFA (18 ± 1 cells, n = 7, P = 0.09) (Figure 6A, B). A microglial inhibitor minocycline (20 μM) and a specific P2X_1,2,3,4_ antagonist TNP-ATP (1 μM), but not the astroglial toxin L-α-AA (1 mM) and a specific P2X_1,2,3,5_ antagonist PPADS (20 μM), decreased the number of cells responding to the ATP in rats subjected to PNI (minocycline: -81 ± 3%, n = 5, P < 0.05, L-α-AA: -20 ± 10%, n = 3, P = 0.3, PPADS: -23 ± 10%, n = 7, P = 0.2, TNP-ATP: -72 ± 4%, *n* = 5, P < 0.05) (Figure 6C). In contrast, the number of cells responding to ATP in rats injected with CFA changed the response to PPADS, but not to minocycline and L-α-AA (minocycline: -5 ± 12%, n = 5, P = 0.2, L-α-AA: -15 ± 12%, n = 3, P = 0.2, PPADS: -71 ± 7%, n = 4, P < 0.01).

## Discussion

In the present study, we examined the contribution of glial cells to central sensitization in the rat spinal dorsal horn which is induced in neuropathic pain and inflammatory pain. We performed immunohistochemical staining of glial cells and imaging of neuronal excitation and ATP-induced Ca^2+^ signaling. In rats subjected to PNI, OX 42 immunoreactivity was markedly increased compared to that in control rats. In rats injected with CFA, GFAP immunoreactivity was increased slightly compared to that in control rats. We showed for the first time that neuronal excitation was augmented in both group of treated rats compared to control rats. Minocycline, SB203580 and TNP-ATP inhibited neuronal excitation in rats subjected to PNI. L-α-AA, carbenoxolone and SP600125 inhibited neuronal excitation in rats injected with CFA. We also showed that the number of cells showing inceased Ca^2+^ signaling in response to ATP was augmented in rats subjected to PNI and the Ca^2+^ signaling was inhibited by minocycline and TNP-ATP.

### Morphological changes of glial cells by inflammatory and neuropathic pain

In agreement with previous studies we showed that the OX 42 immunoreactivity in the ipsilateral spinal dorsal horn increases under the neuropathic pain [7,14], whereas the increase in GFAP immunoreactivity in both group of treated rats was slight. Although Raghavendra *et al.* (2004) reported an increases of OX 42 immunostaining and mRNAs for other markers of microglial activation in the lumbar spinal cord after injection of CFA into the rat hind paw [27], subsequent studies were unable to show an increase of OX 42 immunoreactivity with the same stimulus [28]. In other inflammatory pain models, microglial activation has been observed by immunohistochemistry for OX 42. For example, the perisciatic administration of zymosan, which is a model of sciatic inflammatory neuropathy, significantly increased OX 42 immunoreactivity in the ipsilateral dorsal horn [28]. However, subcutaneous injection of zymosan into the rat hind paw did not induce robust morphological microglial activation [6].

Many studies have reported a slight increase in GFAP immunoreactivity ~1 week after PNI and that it also increases in the chronic period [29]. There are not many studies showing changes in GFAP immunoreactivity in inflammatory pain models, however, after injection of CFA into the rat hind paw, Raghavendra et al. (2004) reported an increase in GFAP immunostaining in the lumbar spinal cord [27], and Gao et al. (2010c) reported an increase in JNK1 in astrocytes bilaterally [30]. Taken together, these observations are consistent with the previous report. That is, pain hypersensitivity in response to nerve injury, but not to inflammation, induces a substantial morphologic change in spinal cord microglia. In contrast, in inflammation, slight morphologic changes in astrocytes occur.

### Hyperexcitability of optically-recorded neuronal excitation by inflammatory and neuropathic pain

Central sensitization was originally described as an immediate-onset, or activity- or use-dependent increase in the excitability of nociceptive neurons in the dorsal horn of the spinal cord via brief nociceptor input. After the induction of central sensitization via a brief intense nociceptor-conditioning stimulus, a mechanism known as subliminal input (which is normally too weak to evoke an action potential in the dorsal horn neurons) begins to activate dorsal horn neurons as a result of increased synaptic efficacy. Central sensitization in the spinal dorsal horn is believed to be a mechanism for the induction and maintenance of pain hypersensitivity [1,2,22,23,31,32].

Voltage-sensitive dyes respond well to the cellular membrane potential. Therefore, optical imaging with these dyes has been used to assess neuronal membrane potential changes and neuronal excitation [21-23]. In the present study, we first showed that the optical responses evoked by single-pulse stimulation to the dorsal root in spinal slices from treated rats were stronger than that in slices from control rats, indicating that excitability in the spinal dorsal horn of treated rats was facilitated, and suggesting that central sensitization in the spinal dorsal horn is a mechanism for maintenance of pain hypersensitivity under conditions of inflammation and nerve injury.

### Contribution of micrglia to the hyperexcitability of neuronal excitation under neuropathic pain

It has been reported that long-term potentiation (LTP), which is a kind of synaptic plasticity and is lasting increase of synaptic strength, in the spinal dorsal horn induced by high-frequency conditioning stimulation (HFS) is inhibited by glial metabolic inhibitor [33,34]. Inhibition of the glutamate transporter 1, which is predominantly expressed by astrocytes, has also been shown to block HFS-induced LTP and c-Fos expression [34]. It has also been reported that the long-term facilitation of optically-recorded neuronal excitation with voltage-sensitive dye induced by low-frequency conditioning stimulation or by ATP incubation in the spinal cord slice is inhibited by blockage of glia-related mechanisms [22,23].

In the present study, we first showed that neuronal excitation in the spinal dorsal horn in rats subjected to PNI was depressed by inhibition of microglia and p38 MAPK and by an antagonist of the P2X_4_ receptor. It has been reported that cells expressing P2X_4_ receptors are microglia and acute pharmacologic blockade of P2X_4_ receptors reverses established tactile allodynia induced by PNI [14].

PNI leads to activation of p38 MAPK in the spinal cord, and acute pharmacologic inhibition of p38 MAPK in the spinal cord suppresses tactile allodynia [30,35]. It has recently been suggested that a release of brain-derived neurotrophic factor from microglia via P2X_4_R-evoked increases in Ca^2+^ and activation of p38 MAPK is a mechanism for pain hypersensitivity after PNI [20]. Our present results suggest that the facilitation of neuronal excitation in the spinal dorsal horn of rats subjected to PNI is mediated by the activation of p38 MAPK and the P2X_4_ receptor in the microglia and that such microglia-related facilitation may be a mechanism for the maintenance of pain hypersensitivity in rats subjected to PNI. However, the specific inhibition of astroglial function by intrathecal injection of fluorocitrate or L-α-AA, or JNK peptide inhibitor has been shown to attenuate pain hypersensitivity in neuropathic pain [4,36]. These results suggest that astrocyte is also important for neuropathic pain. However, it should be emphasized that, as used in the majority of previous studies, we used general glial inhibitors, such as minocycline. Since the mechanism of action remains uncertain, they may have other biological actions or actions to neurons.

### Contribution of astrocytes to the hyperexcitability of neuronal excitation under inflammatory pain

In the present study, we first revealed that the neuronal excitation in the spinal dorsal horn in rats injected with CFA was depressed by inhibition of astrocytes and JNK and by blocking of gap junctions. It has been reported that bilateral activation of JNK in the spinal astrocytes is increased by CFA injection to the rat hind paw, and that mechanical allodynia induced by CFA injection is reversed by pharmacologic blockade of JNK bilaterally [30].

Astrocytes are characterized by the formation of gap junctions that connect them. Connnexin 43 is the major gap junction protein in astrocytes [37]. Although there is no direct evidence showing a contribution of spinal astrocyte gap junctions to pain hypersensitivity in response to CFA injection, some reports indicate a contribution of gap junctions to pain. Hanstein *et al.* (2010) reported that the intraperitoneal gap junction blocker carbenoxolone reverses pain hypersensitivity induced by submandibular CFA injection [38]. In addition, Spataro *et al.* (2004) reported that intrathecal carbenoxolone reverses the bilateral mechanical allodynia induced by the perisciatic injection of zymosan, chronic constriction injury, and intrathecal gp120 [24].

Our present results suggest that the facilitation of neuronal excitation in the spinal dorsal horn of CFA-injected rats is mediated by the activation of astrocyte JNK and gap junctions, and that such astrocyte-related facilitation may be a mechanism for the maintenance of pain hypersensitivity in inflammatory pain in response to CFA injection. However, the intrathecal administration of minocycline has been reported to attenuate formalin-evoked second-phase flinching behaviour or carrageenan-induced hyperalgesia [39]. In the CFA-injected rats, significant increase of microglial markers (Mac-1, TLR4 and CD14) at 4 and 14 days following injection has been reported [27]. These studies suggest that inflammatory pain is also induced by microglia-related mechanisms. Immunohistochemical staining of JNK and connexin will be necessary to identify these mechanisms more directly.

### Augmentation of Ca^2+^ signals evoked by puff-application of ATP via P2X_4_ receptors under neuropathic pain

The measurement of intracellular Ca^2+^ concentration has been used widely to analyze the excitability of glial cells [25,26], particularly in the culture. In the present study, we used the spinal cord slice preparation for Ca^2+^ imaging to examine glial excitability in the context of coexistence with neurons. In the slice preparation, ATP can induce Ca^2+^ influx not only in glial cells but also in neurons and primary afferent terminals in the spinal dorsal horn [40]. The Ca^2+^ signals observed in the present study may represent a mixture of glial cells, neurons and afferent terminals. However, the Ca^2+^ signal in rats subjected to PNI was largely inhibited by minocycline and a specific P2X_1,2,3,4_ antagonist TNP-ATP (1 μM), but not by a P2X_1,2,3,5_ antagonist PPADS (20 μM). Therefore, it is likely that the increase in cells showing a Ca^2+^ response to ATP in rats subjected to PNI in comparison to control and CFA-injected rats reflect P2X_4_ receptors in spinal microglia. Identification of glial cells in spinal cord slices will be necessary to further clarify this.

## Conclusions

In the present study, we have shown that hyperexcitability in the spinal dorsal horn under the condition of pain hypersensitivity induced by nerve injury or inflammation is mediated by microglia-related and astrocyte-related mechanisms, respectively. Our results suggest that glia-mediated central sensitization in the spinal dorsal horn is a mechanism for pain hypersensitivity and that there are distinct plastic changes in glia-neuron interactions in different forms of persistent pain.

It should be emphasized that we used young rats in the present study. It has been reported that the development of glial responses and allodynia to nerve injury differs according to age in rats [41,42]. In addition, we examined only a 1-week period after injury and inflammation. It has been reported that astrocytes are activated during the chronic period after PNI [35]. Therefore, it is possible that the astrocyte-mediated hyperexcitability observed in the present study might also occur during the chronic period after PNI. Further investigations are necessary to reveal the developmental changes.

## Methods

### Animal models

All animal studies were undertaken according to protocols approved by the university animal ethics committee in University of Fukui on 20- to 30-day-old male and female Wistar rats, weighting 45-70 g. For the PNI model, the left spinal L5 nerve root was ligated under 2,2,2-tribromoethanol anesthesia (0.2 g kg^-1^, intraperitoneally, Wako Pure Chemical Industries). For the CFA (inflammatory) model, 50 μl CFA (Sigma-Aldrich) was injected into the left intraplantar hind paw. Mechanical allodynia was assessed by determining foot withdrawal to 50% of mechanical thresholds after mechanical plantar stimulation with von Frey filaments (North Coast Medical) according to an up-down paradigm [43].

### Slice preparation

Rats were anaesthetized with diethyl ether. After laminectomy, the spinal cord was excised, and several transverse slices (400-μm thick) with dorsal roots attached were prepared from the lumbosacral enlargement (L4-6). The rats were then killed with an over dose of ether.

### Membrane potential imaging

Each slice was stained in a bath filled with the voltage-sensitive absorption dye, RH-482 (0.1 mg ml^-1^, 20 min), and set in a submersion-type chamber (0.2 ml) on an inverted microscope (IMT-2, Olympus) equipped with a 150-W halogen lamp. Slices were perfused with Ringer solution containing (in mM): 124 NaCl, 5 KCl, 1.2 KH_2_PO_4_, 1.3 MgSO_4_, 2.4 CaCl_2_, 26 NaHCO_3_, 0.2 thiourea, 0.2 ascorbic acid, and 10 glucose (oxygenated with 95% O_2_ and 5% CO_2_) at room temperature (23 ± 2 °C). The change in light absorption, at a wavelength of 700 ± 32 nm in a 0.83-mm^2^ area of the dorsal horn was recorded with an imaging system (Deltalon 1700, Fuji Film) equipped with 128 × 128 pixel photo sensors at a frame rate of 0.6 ms. The dorsal root was stimulated by a current pulse of 2 mA with a duration of 0.5 ms through a glass suction electrode. Sixteen single pulses were administered at a constant interval of 15 s. Starting at 10 ms before each stimulus, the image sensor acquired 128 consecutive frames of light-absorption images at a sampling interval of 0.6 ms. A reference frame, which was acquired immediately before each series of 128 frames, was subtracted from the subsequent 128 frames. Sixteen series of such difference images were averaged and stored. We determined the initial frame by averaging the first 15 frames of the difference image and then subtracting this average from each of the 128 frames of image data on a pixel-by-pixel basis to eliminate the effects of noise in the reference frame. The ratio image was then calculated by dividing the image data by the reference frame.

### Ca^2+^ imaging

Each slice was loaded for 45 min at room temperature with 10 μM Fluo-4/AM in the presence of 0.01% Pluronic F-127 (Molecular Probes). Slices were then washed thoroughly with Ringer solution, and set in a submersion-type chamber (0.2 ml) on an inverted microscope (IX71, Olympus). Confocal images of Fluo-4 fluorescence were captured at 1 frame s^-1^ with a CSU10 Nipkow spinning-disk confocal microscope (Yokogawa Electric), equipped with an EM CCD camera (iXON EM, Andor). Fluo-4 fluorescence was excited by light at 488 nm from a semiconductor laser. ATP was puff- applied for 5 s at 20 s after the start of recording.

### Immunohistochemistry

Rats were deeply anesthetized and perfused intracardially with 0.01 M phosphate-buffered saline (PBS) followed by 4% cold, buffered paraformaldehyde. The spinal cord was then removed immediately, postfixed at 4 °C overnight in the same fixative and then cryoprotected in 20% sucrose in PBS (pH 7.4) for 48 h and sectioned at 40-μm thickness. Nonspecific antibody binding was inhibited by incubating the slices in 3% normal goat serum. Slices were then incubated for 48 h at 4 °C with antibody against OX42 (anti-OX 42, 1:400, Serotec), a microglia marker, or with antibody against glial fibrillary acidic protein (GFAP) (anti-GFAP, 1:400; Sigma-Aldrich), an astrocyte marker. After incubation, tissue sections were washed and incubated for 3 h at room temperature with secondary antibody solution (anti-rabbit IgG-conjugated Alexa Fluor 488, 1:400; Molecular Probes). Immunofluorescence intensity measurements were obtained with a digital camera (DP72, Olympus) on a fluorescence microscope (AX80, Olympus). For the image analysis, the free image analysis software ImageJ 1.40f (NIH) was used to measure the density of OX 42- or GFAP-immuoreactivity. Quantitative assessment was made by determining the immunofluorescence intensity within a fixed area of the central substantia gelatinosa (200 μm^2^), and the mean intensity of this area recorded. This protocol was carried out on 3 L4-6 spinal sections from each rat. The background fluorescence intensity of each section was also determined and subtracted from values obtained.

### Statistical analysis

Results are expressed as means ± standard error of the mean (SEM). The Student’s paired t-test was used to assess statistical differences.

## Competing interests

The authors declare that we have no competing interests.

## Authors' contributions

HI and TK performed experiments and analyzed data. HI conceived and designed the study. HI and KM wrote the manuscript. All authors have read and approved the final manuscript.
